# The Utility of Nucleolar Organizer Regions Quantitation in Early Prediction of Lung Neoplastic Transformation

**DOI:** 10.7759/cureus.11738

**Published:** 2020-11-28

**Authors:** Hussain G Ahmed, Amel B El Hag, Naif K Binsaleh, Gamal Eldin Mohamed O Elhussein, Malik Asif Hussain, Mohamed Ahmed Babikir I Bealy, Emad Abboh A Abboh, Hisham Sherfi

**Affiliations:** 1 Pathology, College of Medicine, University of Ha'il, Ha'il, SAU; 2 Pathology, College of Medicine, University of Ha’il, Ha’il, SAU; 3 Clinical Laboratory, College of Applied Medical Science, University of Ha’il, Ha’il, SAU; 4 Pediatrics, College of Medicine, University of Ha’il, Ha’il, SAU; 5 Medicine, College of Medicine, University of Ha’il, Ha’il, SAU

**Keywords:** agnors, nor, lung cancer, cigarette smoking, saudi arabia

## Abstract

Background: Cancer burden can be reduced by early detection of early neoplastic changes applying suitable screening methods. This study aimed to assess the utility of nucleolar organizer regions (NORs) quantitation in early prediction of lung neoplastic transformation.

Methodology: This study investigated 200 apparently healthy individuals categorized into two groups; smoking exposed individuals (N=100), and were categorized as cases, and smoking nonexposed (N=100), and were ascertained as controls. Sputum specimen was attained from each participant (paying all indispensable safety precautions and sample adequacy processes).

Results: Out of the 200 volunteers assessed in the present study, mean NORs counts of >2.00 were identified in 16/200(8%) of the study subjects. All 16/16(100%) cases were found with lung epithelial metaplasia (squamous metaplasia). Out of the 100 cases, mean NORs counts of >2.00 were identified in 16/100(16%), hence, all the controls were identified with mean NORs counts of <2.00. The risk of lung cellular proliferative changes associated with smoking exposure are odds ratio (OR) (95% confidence interval, CI) = 39.2485 (2.3199-664.0052), p = 0.0110, z statistic = 2.543.

Conclusion: NORs count is a simple, specific, cost-effective, and reliable method that can give a quantitative measurement for the risk of lung neoplastic transformation. For at risk-population (tobacco users), it is recommended to perform the argyrophilic NORs (AgNORs) method beside sputum cytology.

## Introduction

Over 85% of lung cancer cases have been linked to smoking [1]. Smoking causes about 1.7 million causalities per annum globally [2]. Early detection of carcinoma is important from a therapeutic point. Late-stage diagnosis of lung cancers is associated with very high mortality compared to early diagnosis [3]. Lung cancers have the least survival rate for five years amongst all malignancies [4]. Lung cancer screening has been reported to reduce mortality [5]. The screening and smoking cessation have even more reduction in the death rate [6]. A bonus benefit of screening is the opportunity to encourage and engage smokers in quitting programs [7]. The people at higher risk of having lung malignancy based on their age and history of smoking are recommended to get screening for lung cancers [8].

Nucleolar organizer regions (NORs) are loops of DNA that have encoding for rRNA and play an important role in protein synthesis in cells. They chemically bind with silver, the complex formed is referred to as argyrophilic NOR (AgNOR) which is observed to count their numbers [9].

DNA damage related changes in NORs are important [10]. The NORs numbers can be used as an important marker in tumor studies [11]. Malignant lesions possess NORs in higher numbers and this increased number is also linked with poor prognosis. Their number reflects the cellular division activity as well as dysplasia in many tissues [12]. Their number can even be used to study the effects of pollutants or therapy on cell kinetics [13]. A higher number of NORs can be used to decide treatment options such as whether the systemic therapy for lung cancer patients is required or not. There is a difference in NORs at various stages and in different types of lung cancers. For example, NORs can be used to monitor the prognosis and recurrence of nonsmall cell lung cancers [14].

Recent research is focusing on revealing further genomic details about human NORs. It is important to investigate the relationship between various diseases as well as the aging process and NORs [10]. As the AgNOR mean count is a noninvasive, cost-effective procedure, the raised question is whether it is the most suitable simple method for screening at risk population, such as smokers. Current diagnostics and treatment procedures need further research. Therefore, the current study aimed to assess the utility of NORs quantitation in early prediction of lung neoplastic transformation.

## Materials and methods

In this case-control study, 200 apparently healthy volunteers were randomly selected by simple random method regardless of their demographical characteristics. Study subjects were categorized into two groups; smoking exposed individuals (N=100), and were categorized as cases, and smoking nonexposed (N=100), and were ascertained as controls. Sputum specimen was attained from each participant (paying all indispensable safety precautions and sample adequacy processes). Cellular constituents were cautiously selected from the colored sputum matters and then were evenly smeared on a cleaned frosted-end glass-slide. Each smear was immediately fixed in 95% ethyl alcohol for 15 minutes. The smears were then transported to the histopathology laboratory at the College of Medicine, University of Ha’il, Saudi Arabia for staining and diagnosis. All smears were stained using the AgNORs staining method adopting the procedure described by Ahmed and Babiker [15].

Ethical consent

Each participant was asked to sign a written ethical consent before the taking of the biological specimen. The protocol for the study was also approved by the Ethical Committee at the College of Medicine, University of Ha’il. Ethical Approval Number: HREC 00121/CM-UOH.04/20.

Data analysis

All obtained data were entered via a computer software SPSS and analyzed to obtain the Chi-square test (p-value < 0.05 considered statistically significant), odds ratio (OR), applying a 95% confidence interval (CI).

## Results

**Figure 1:**
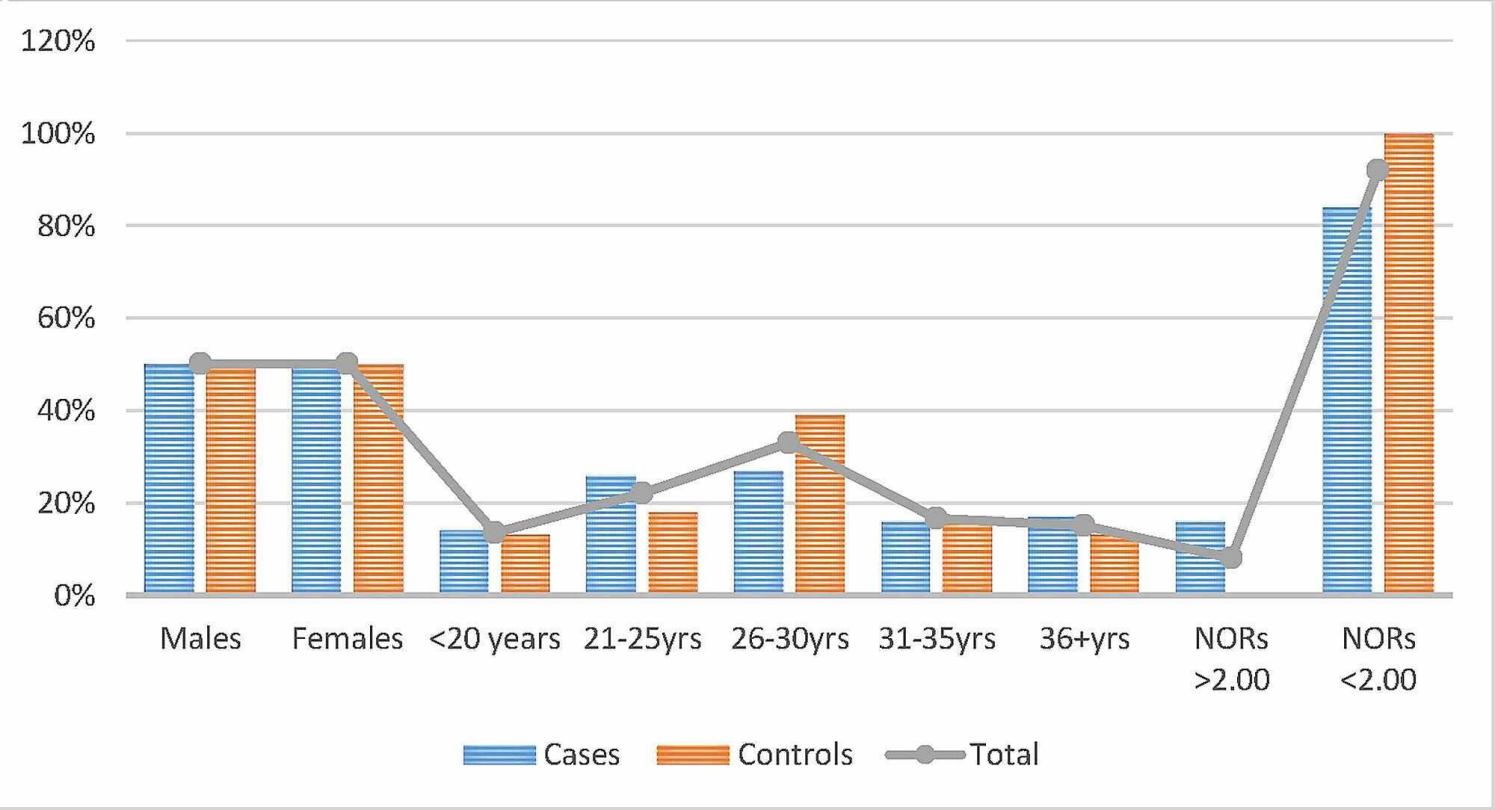
Description of the cases and controls by age, sex and mean NORs counts. NORs, nucleolar organizer regions

**Figure 2:**
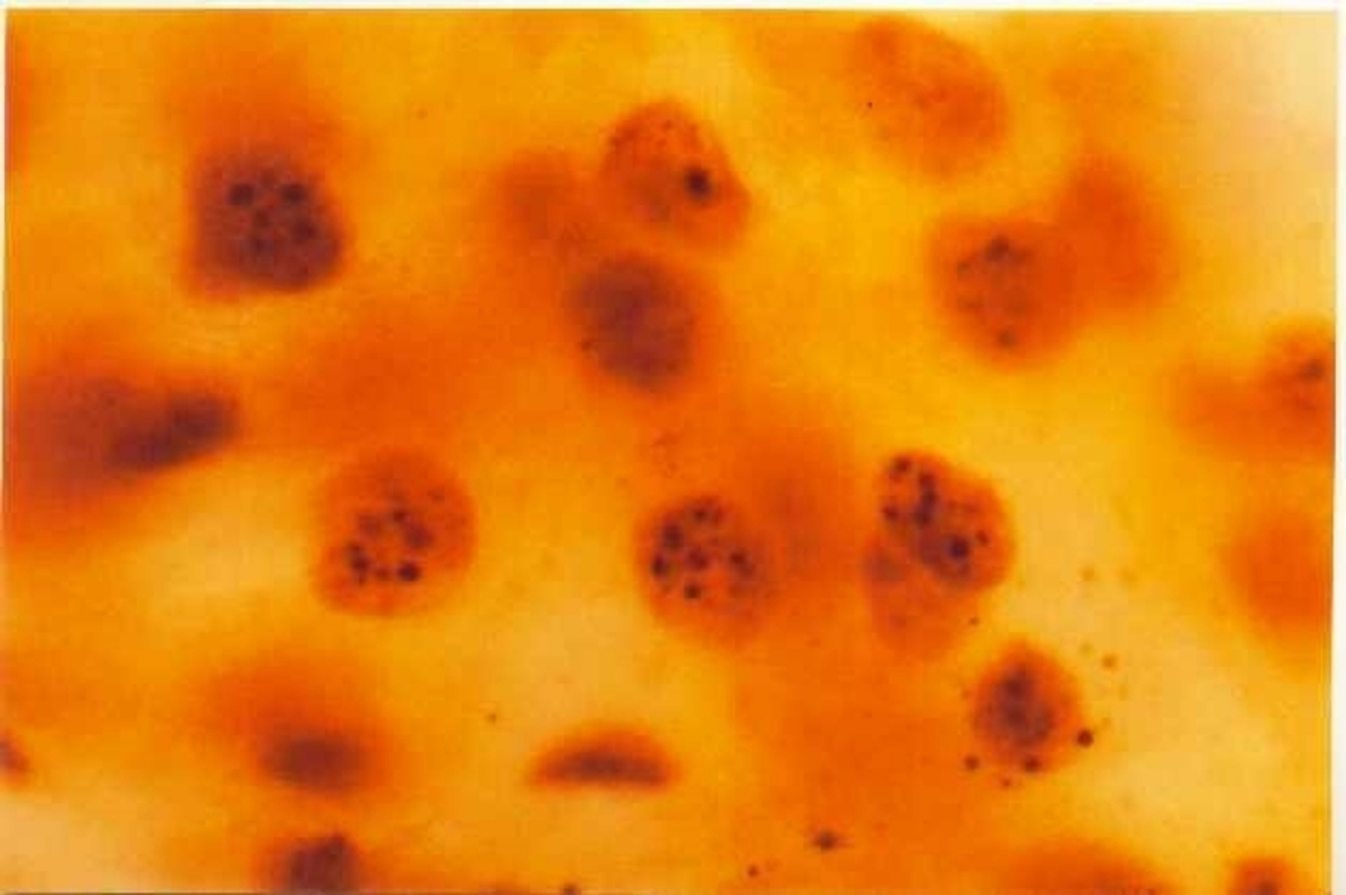
AgNOR stained sputum obtained from individuals exposed to smoking (case). The smears showing mean NORs counts of >2.00 (black dots within the nuclei of the cells). AgNOR, argyrophilic NORs; NORs, nucleolar organizer regions

**Figure 3:**
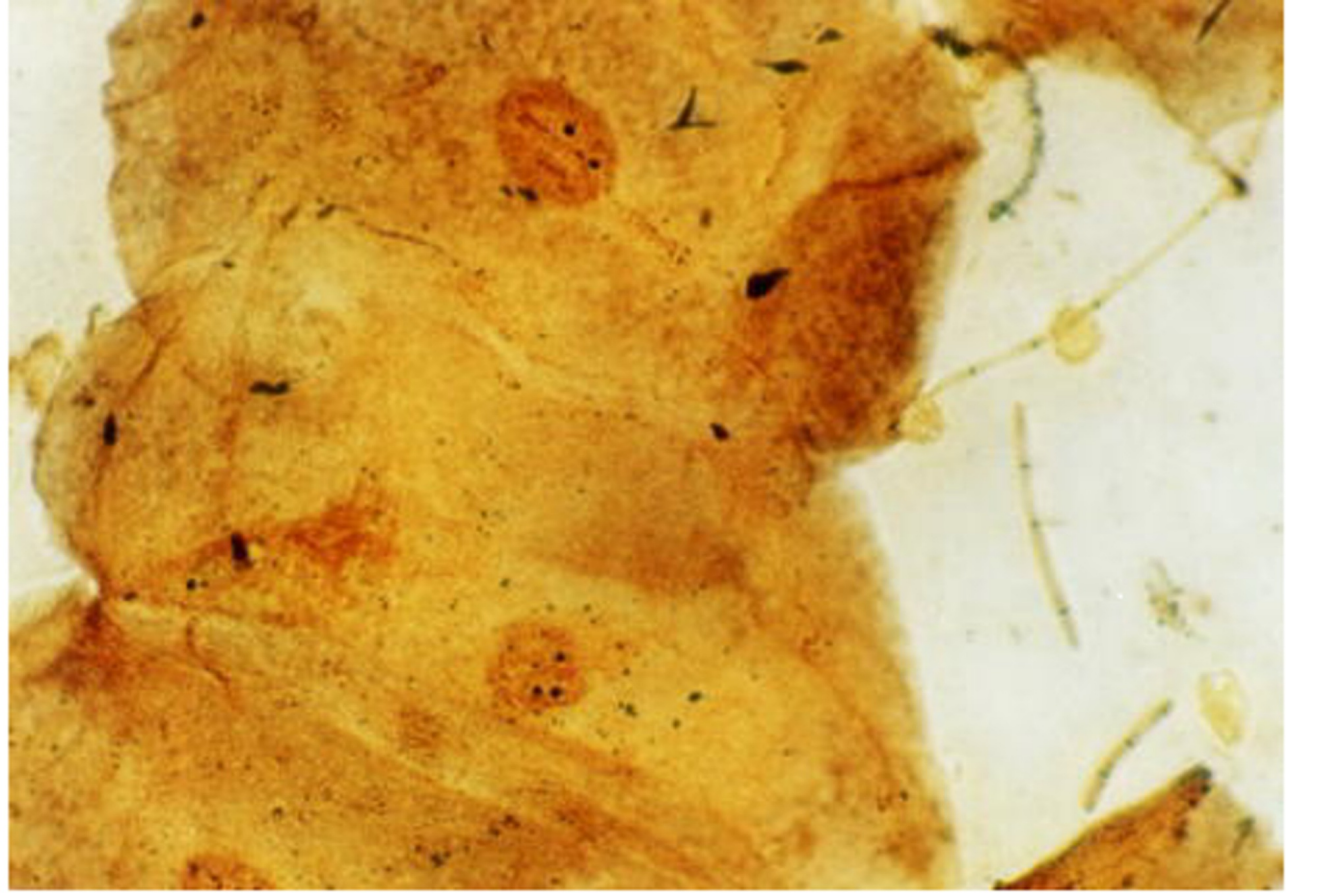
AgNOR stained sputum obtained from individual nonexposed to smoking (control). The smears showing mean NORs counts of <2.00 (black dots within the cells' nuclei). AgNOR, argyrophilic NOR; NORs, nucleolar organizer regions

**Figure 4:**
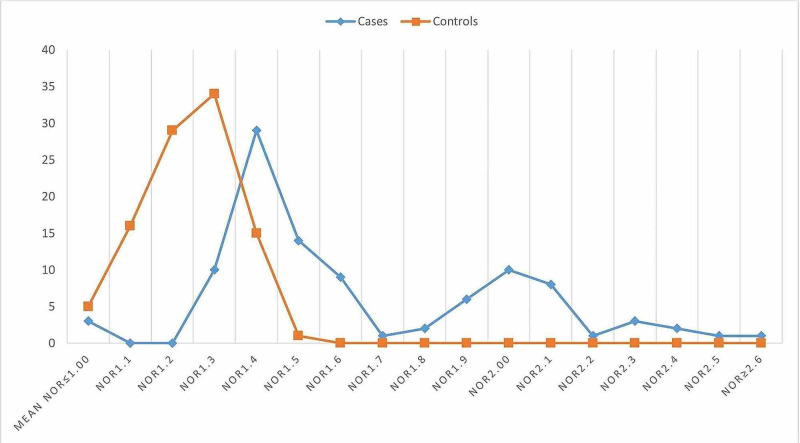
Description of the study subjects by mean NORs counts. NORs, nucleolar organizer regions

**Figure 5:**
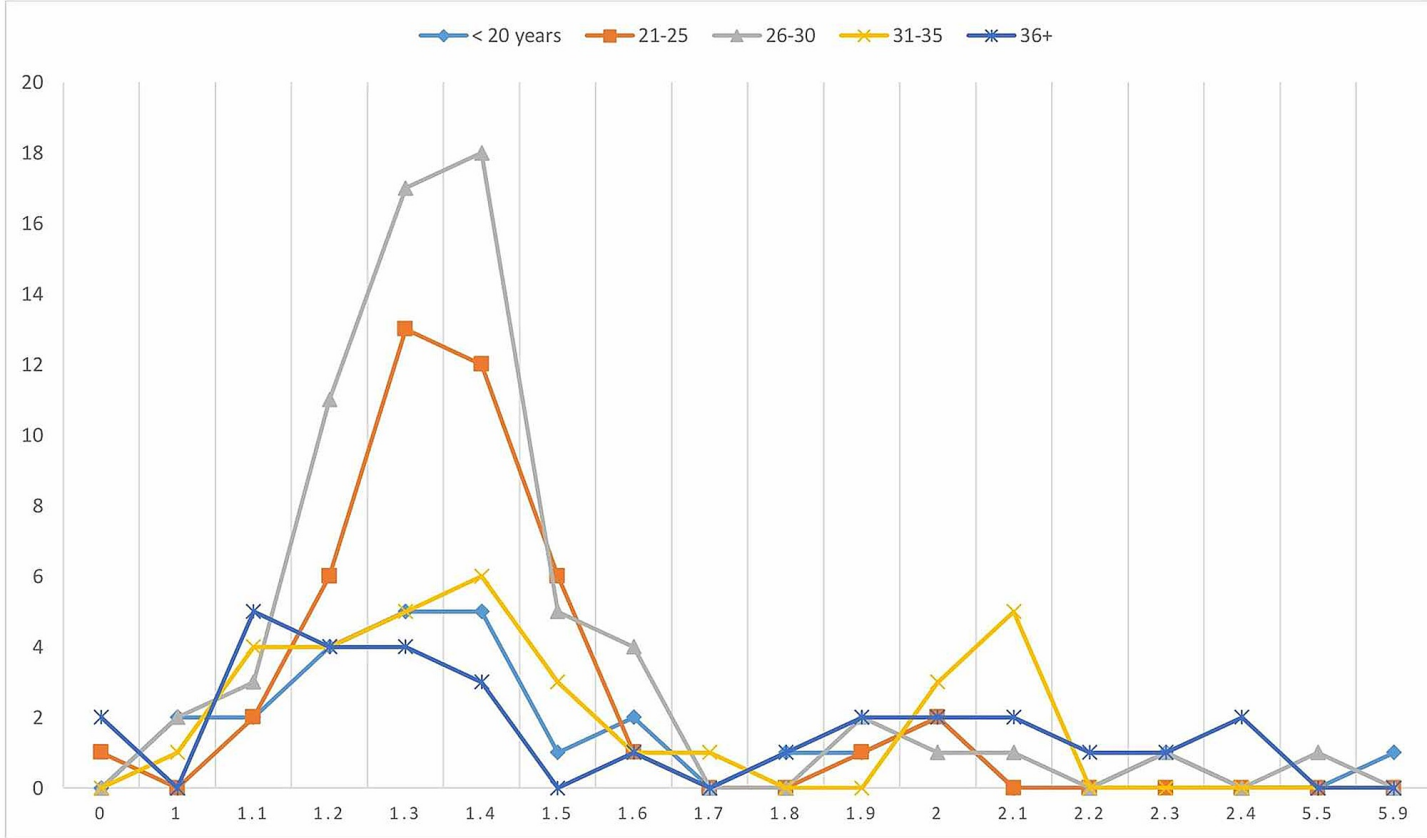
Description of the study population by age and mean NORs counts. NORs, nucleolar organizer regions

**Figure 6:**
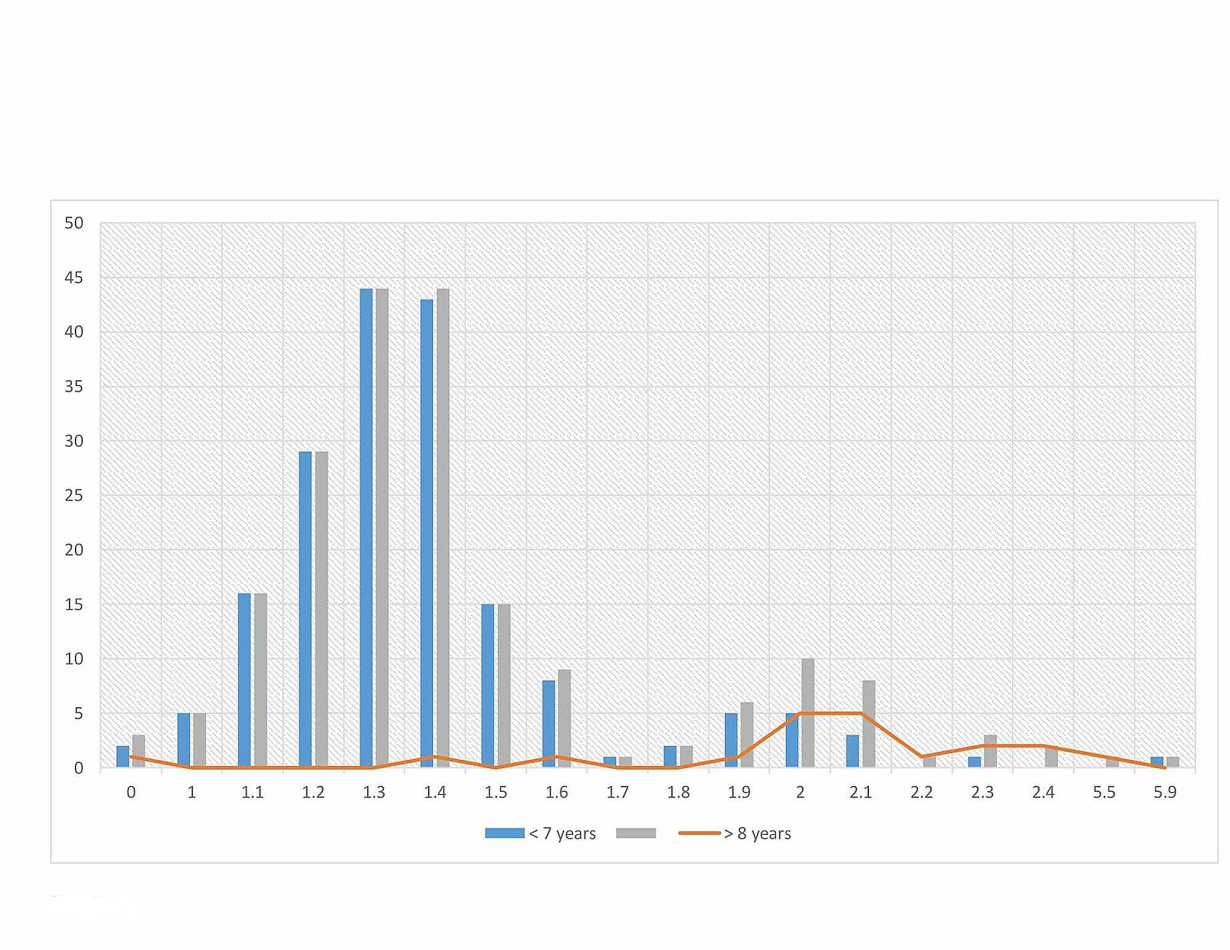
Description of the study subjects by mean of NORs counts and duration of exposure to smoking exposure. NORs, nucleolar organizer regions

The age and sex distribution were relatively similar in the present study, as indicated in Table 1 and Figure 1. Out of the 200 volunteers assessed in the present study, mean NORs counts of >2.00 were identified in 16/200(8%) of the study subjects as shown in Figures 2-3, and the remaining 184/200(92%) were found with mean NORs counts of <2.00. All 16/16(100%) cases were found with lung epithelial metaplasia (squamous metaplasia). Out of the 100 cases, mean NORs counts of >2.00 were identified in 16/100(16%), hence, all the controls were identified with mean NORs counts of <2.00. The risk of lung cellular proliferative changes associated with smoking exposure and the OR and 95% CI was; OR (95% CI) = 39.2485(2.3199-664.0052), p = 0.0110, z statistic = 2.543, as indicated in Table 1, Figures 1, 4. The 16 individuals with mean NORs counts of >2.00 were males. Higher mean NORs counts relatively increased with the increase of age, and duration of exposure to tobacco smoking, as shown in Figures 5-6.

**Table 1:** Distribution of the cases and controls by age, sex, and mean NORs counts. NORs, nucleolar organizer regions

Variable	Cases	Controls	Total
Sex			
Males	50	50	100
Females	50	50	100
Total	100	100	200
Age range			
<20 years	14	13	27
21-25	26	18	44
26-30	27	39	66
31-35	16	17	33
36+	17	13	30
Total	100	100	200
Mean NORs counts			
>2.00	16	0	16
<2.00	84	100	184
Total	100	100	200

 

## Discussion

The finding of a simple, specific, and cost-effective method for early detection of cancerous and precancerous conditions represents an emergent need for continuous screening of at-risk populations. Sputum cytology is a suitable method for screening of people at risk of developing lung cancer, but it has some limitations as it depends on qualitative measures. AgNOR as a quantitative method can be a suitable counterpart filling limitation in traditional cytology. Therefore, the current study aimed to assess the utility of NORs quantitation in early prediction of lung neoplastic transformation.

It is well documented that lung exposure to tobacco smoking has diverse pathological effects including lung neoplastic transformation [16].

In the present study, all cytological smears showing lung squamous metaplasia were found with mean NORs count >2.00, which indicates the merit of AgNORs count in the prediction of lung neoplastic transformation and therefore, suitable as a screening tool for an at-risk population. Higher NORs count usually an indication for cellular proliferation, which is considered as an initial step towards neoplastic progression [17]. These nuclear regions are associated with rDNA which usually indicates cellular activity, mostly related to cell division [18].

AgNORs count method was widely used in oral exfoliative cytology particularly among cigarette smokers to predict early cellular proliferative activity indicating neoplastic change [19], but its use for lung cytology is scarce [20]. It was reported that cellular proliferative quantified by AgNORs might play a substantial role in the early detection and diagnosis of premalignant and malignant lesions in the absence of clinical manifestations [21].

It was well established that the AgNORs quantification method is useful for early detection of cigarette smoking carcinogenic effects on the oral epithelial cells [22]. However, the utility of AgNORs count was not being applied in lung cancer in recent years, though some ancient reports have documented the relationship. The NORs have been utilized in nonsmall cell lung cancer associated with clinical and pathological parameters [23].

On the other hand, the role of cigarette smoking in the etiology of lung neoplastic transformation is well documented [24-25]. Cigarette smoking is prevalent in Saudi Arabia, especially amongst the younger generation [26], which necessitates urgent tobacco prevention control measures at community levels.

## Conclusions

Nucleolar organizer regions count is a simple, cost-effective, and reliable method that can give a quantitative measurement for the risk of lung neoplastic transformation. For at risk-population (tobacco users), it is recommended to perform the AgNORs method beside sputum cytology.
